# Review on Bubble Dynamics in Proton Exchange Membrane Water Electrolysis: Towards Optimal Green Hydrogen Yield

**DOI:** 10.3390/mi14122234

**Published:** 2023-12-12

**Authors:** Bongliba T. Sangtam, Hanwook Park

**Affiliations:** Department of Biomedical Engineering, Soonchunhyang University, 22 Soonchunhyang-ro, Asan 31538, Chungnam, Republic of Korea; drsangtam@sch.ac.kr

**Keywords:** PEM water electrolysis, bubble dynamics, hydrogen evolution reaction, oxygen evolution reaction, two-phase flow

## Abstract

Water electrolysis using a proton exchange membrane (PEM) holds substantial promise to produce green hydrogen with zero carbon discharge. Although various techniques are available to produce hydrogen gas, the water electrolysis process tends to be more cost-effective with greater advantages for energy storage devices. However, one of the challenges associated with PEM water electrolysis is the accumulation of gas bubbles, which can impair cell performance and result in lower hydrogen output. Achieving an in-depth knowledge of bubble dynamics during electrolysis is essential for optimal cell performance. This review paper discusses bubble behaviors, measuring techniques, and other aspects of bubble dynamics in PEM water electrolysis. It also examines bubble behavior under different operating conditions, as well as the system geometry. The current review paper will further improve the understanding of bubble dynamics in PEM water electrolysis, facilitating more competent, inexpensive, and feasible green hydrogen production.

## 1. Introduction

Over the years, global energy consumption has risen sharply, primarily because of population growth and increased living standards. The need for substituting fossil fuels with clean energy is urgent due to global warming and growing environmental issues. It has been predicted that the amount of energy generated from renewable sources will rise by 2.3% by 2040, accounting for 31% of all electricity produced globally [1]. The Paris Agreement Act mandates that the increase in world temperature needs to drop below 2 degrees by 2050 by adopting the green hydrogen revolution for sustainable energy for the decarbonization process to combat global warming [2]. Although different methods are available to produce hydrogen, the one that is derived from renewable resources is gaining momentum as a cleaner energy source that could substitute for conventional fossil fuels [3]. Compared to other clean energy sources, hydrogen tends to be greener, and it creates negative carbon as a byproduct [4]. Water electrolysis has been proven to be more dependable than traditional methods of hydrogen production, offering a high level of safety, more sustainability, and a purity of up to 99.99% [5]. Hydrogen is widely used in conventional industries such as petroleum, petroleum derivatives, and chemical fertilizers [6]. As a result of recent progress in research and development on electric vehicles powered by fuel cells that discharge zero carbon emissions, the demand for hydrogen has substantially increased [7]. With continued scale production, the price of green hydrogen produced using water electrolysis has been forecasted by CSIRO to become competitive with thermochemical processes by 2025 [8]. The supply of sustainable hydrogen has been limited by the high cost of infrastructure setting [9]. However, with the aid of cutting-edge technology, it can be much enhanced in design, which will undoubtedly make a beneficial impact on the water electrolysis process to harvest more renewable energy [10].

**Figure 1:**
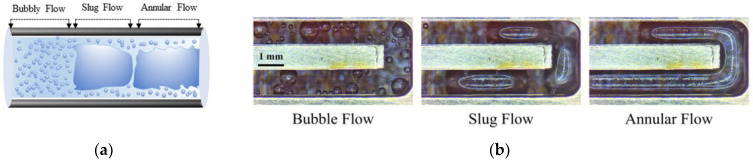
Flow patterns in PEM water electrolysis channel. (**a**) Different gas bubble formation. (**b**) Observation of different gas bubble flow patterns inside the cell. Copyright 2023, with permission from Elsevier [20].

Water can be electrolyzed using different approaches, including alkaline water electrolysis (AWE) [11], anion exchange membrane water electrolysis (AEM) [12], proton exchange membrane water electrolysis (PEM) [13,14], and solid oxide electrolysis (SOE) [15]. PEM water electrolysis has been shown to be more cost-effective than the other techniques. It can also work at higher current densities, whereas others are more prone to rapid changes in the current load. Furthermore, PEM can operate substantially faster than AWE and SOE, which take longer time for operations [16]. In the PEM water electrolysis, water is separated as oxygen and hydrogen through electrochemical processes. Water is supplied from the anode side and then it moves between the catalyst layer and the liquid/gas diffusion layer, thus reacting with the catalyst, resulting in the breaking of water into oxygen, proton, and electron [17]. Protons then leave the membrane and fuse with the electrons from the applied current density to create hydrogen on the cathode side, while gas bubbles simultaneously enter the flow field on the anode side [18]. On the anode side, the solvated proton migrates to the cathode side, and it is accompanied by a water molecule that flows from the anode to the cathode side region. As a result, even in the absence of water from the anode during the PEM electrolyzer operation, the PEM remains hydrated [19]. The various flow patterns in the PEMWE channel are shown in Figure 1a,b [20].

**Figure 2:**
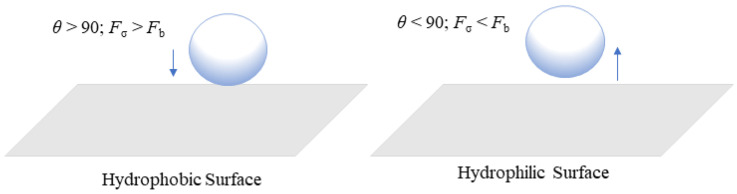
Formation of bubbles at hydrophobic and hydrophilic surfaces.

For flexible use, it is critical to address these challenges by increasing current densities and system efficiency to reduce investment costs and broaden the range of uses for this innovation [21]. The formation of gas bubbles at the catalyst layer in the anode region is one of the key issues. It can interrupt effective contact between the catalyst and water, decreasing the electrochemical reaction on the anode side [22]. Thus, it is extremely important to select a highly efficient catalyst for faster removal of gas bubbles from the system [23]. When gas produced by the catalyst exceeds the capacity of flow channels, a bubble blockage may occur. This can be estimated based on the cross-sectional area and water flow rate of channels [24]. Studying bubbles is crucial in proton exchange membrane water electrolysis (PEMWE) because when bubbles develop at the catalyst layer, they can obstruct tiny pores and restrict water flow, which can increase equipment costs and affect performance efficiency [25,26]. Furthermore, when the bubble separates from the electrode surface, the empty area formed by the prior bubbles gets filled, resulting in a swirling motion [27]. The growth of bubbles inside tiny pores can induce a pressure drop, which can cause considerable mechanical stress on the catalyst surface (CS), resulting in the gradual deterioration of a catalyst structure [28]. Gas bubbles generated from the CS will pass through the liquid/gas diffusion layer (LGDL) and eventually enter the flow channel, thus creating two-phase flows such as bubbly, slug, and annular flows [29]. When the applied current density is lower, bubble coalescence occurs at a low frequency, resulting in smaller bubbles within the channel, and this flow is considered as bubbly flow [30]. As the current density increases, bubbles combine more frequently, forming a slug. As gas density increases, the slug develops into an annular flow regime. The gas phase then occupies almost the entire channel length, forcing water toward the channel wall [17]. The various flow patterns in the PEMWE channel are shown in Figure 1a, b. These flow regimes depend on different factors such as mass flux and superficial velocity of liquid and gas phases [31]. The flow pattern has a significant impact on water management and distribution because it controls how the reactant and product travel throughout the electrolysis operation. In PEM water electrolysis, the channel wall must be kept wet to prevent the degradation of the membrane in the cell. The transaction from annular to mist flow can result in insufficient liquid wet on the channel walls and it can cause a high risk of damage to the membrane [29]. Chien and Ibele [32] calculated this value as 1.199 × 10^6^ to predict the transaction from annular to annular–mist flow in two-phase flow systems. This criterion value was developed for the vertical flow in larger pipe diameters, but this can also be used for predicting when the flow regime shifts from annular to mist flow in PEM water electrolysis [29]. The efficiency of the electrolysis system depends on how fast the gas bubbles are controlled and removed from the membrane surface and the flow channels. Figure 2 shows how bubbles are formed in the catalyst layer based on hydrophilic and hydrophobic surfaces. The formation of bubbles on the hydrophilic surface remains spherical [33]. Jiang et al. [34] have studied how different combinations of contact angles at the PTL and catalyst layer can impact cell performance at a constant voltage of 2 V. For the dividing line between hydrophilic and hydrophobic surfaces, they used a contact angle of 90°. They found that the catalyst layer with a hydrophilic surface was 12.6 times higher than that with a hydrophobic surface. The main reason for this finding is that in a hydrophilic condition, the volume of gas concentration within the catalyst layer is low, which can reduce the bubble effect and hence mass transfer losses. This has assisted in understanding that the electrochemical reaction occurs not only on the catalyst layer (CL) but also at the CL–LGDL contact [35]. Understanding the behavior of bubbles at CL–LGDL will provide further details about how bubbles develop, grow, and detach from a cell. With the aid of this knowledge, the distribution of a catalyst and the design of a cell may be enhanced, which can increase the efficiency of the electrochemical process inside the cell.

During the process of electrolysis, bubbles can generate motion in the surrounding liquid, which can improve mixing and mass transfer rates [27,36]. Identifying how bubbles behave will help a cell function better, allowing for the detection of any detrimental effects on the system and the development of new, innovative electrochemical technologies that will lead to more sustainable and effective energy [37]. The different operating conditions such as current density, temperature, and water flow rate can also impact the stability of the PEMWE system. Based on PEMWE modeling, it has been established that the performance of a cell is dependent on the amount of water input and that both temperature and liquid flow rate can affect current density [38,39]. As the liquid flow rate increases, larger bubbles will disperse into smaller sizes, resulting in a reduction in slug flow. However, as current density and temperature increase, larger bubbles and longer slugs will form inside the cell. With an increase in current density, a substantial number of bubbles will amalgamate, resulting in the production and wide distribution of gas bubbles. This causes bubbles to migrate toward their larger neighbors, resulting in rapid growth [40]. When flow velocity increases, bubbles begin to move faster, causing large slug gas to split up and move along the flow velocity. Li et al. [41] and Ojong et al. [42] have shown that a higher liquid velocity on the anode side can facilitate bubble separation, thus reducing mass transport loss. Therefore, understanding bubble behavior is critical for improving the mobility of this process. This understanding will aid in the development of more effective and efficient electrochemical systems for the PEM electrolysis process.

In this review paper, the core principles of PEM water electrolysis are outlined along with a comparison with PEM fuel cells. It also highlights various materials utilized for constructing PEM water electrolysis, especially the selection of appropriate materials, such as current distributor plates, porous transport layers, and catalyst-coated membranes. The most significant focus of this article is based on the role of the dynamics of gas bubbles in PEM water electrolysis, concentrating on how bubbles nucleate, grow, and detach during the process. This study also examines various losses caused by the instability of bubbles on the electrode surface, such as activation, ohmic, and diffusion. An in-depth study of bubble dynamics in flow channels, catalyst layers, and PTLs is conducted. Different techniques available to capture bubble images are also presented. The outcomes of this research will aid in improving bubble management in PEMWE and the effectiveness and scalability of green hydrogen production by water electrolysis.

## 2. Basic Principle of PEM Water Electrolysis and PEM Fuel Cells

The working mechanism of PEM water electrolysis is the same as that of PEM fuel cells as both use solid membranes to exchange protons between anode and cathode sides. However, their purposes and directions of electrochemical processes differ. In PEM water electrolysis, electric energy is used to split water into oxygen and hydrogen to produce hydrogen gas toward the cathode. However, in a PEM fuel cell, electricity is generated by introducing hydrogen and oxygen from both channels with water being the only byproduct [43]. When hydrogen gas enters the anode catalyst layer, it splits into two parts. One part generates a proton (H^+^), which travels to the cathode via the proton exchange membrane. The other element is converted into an electron (e^−^), which travels via an external circuit and offers an electric current [44]. In a PEM water electrolysis, the method involves supplying electrical current within, which includes the end plate, bipolar plate, GDL, MEA, and catalyst layer. Table 1 presents the advantages and disadvantages of PEM water electrolysis and PEM fuel cells. When water is introduced into the anode region, it undergoes oxidation and forms oxygen, hydrogen ions, and electrons, which is shown in Equation (1). After splitting water molecules (H_2_O) into individual parts, ions with a positive charge (H^+^) will react with water molecules to generate hydrated hydrogen ions (H^+^. *x*H_2_O) [45]. These hydrated hydrogen ions subsequently move through the proton exchange membrane and enter the cathode (Equation (2)) to produce hydrogen. The overall reaction is presented in Equation (3) [46].

(1) $$\mathrm{Anode}\;\mathrm{side}:\;{2H}_{2}O\;\to {\;O}_{2}+{4H}^{+}{+4e}^{-}$$

(2) $$\mathrm{Cathode}\;\mathrm{side}:\;4H^{+}+{4e}^{-}\to 2H_{2}$$

(3) $$\mathrm{Overall}:\;H_{2}O\to H_{2}+\frac{1}{2}O_{2}$$

**Figure 3:**
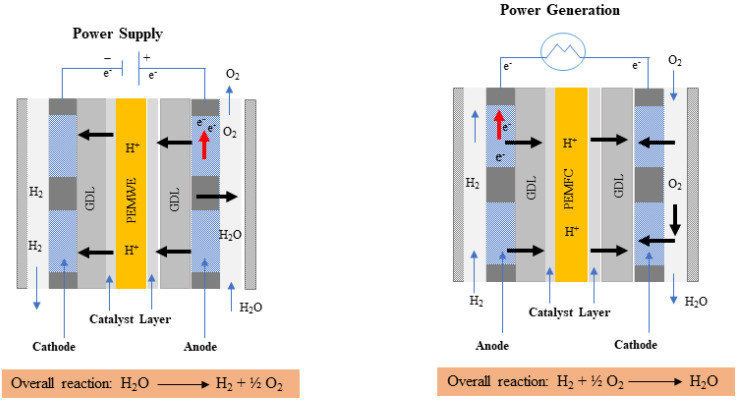
Schematic diagram of a PEM water electrolysis and PEM fuel cell.

Contrary to the conventional approach, which requires an electrolyte solution, PEM water electrolysis uses a simple proton exchange membrane. Figure 3 presents a schematic illustration of the PEM of the water electrolysis and fuel cell. The thin membrane would allow the positively charged particle to move freely, minimizing the restriction on mass transfer. Additionally, the electrolyzer process uses clean water rather than the substantial electrolyte solutions necessary for optimal electronic conductivity.

### Materials of Construction

**Figure 4:**
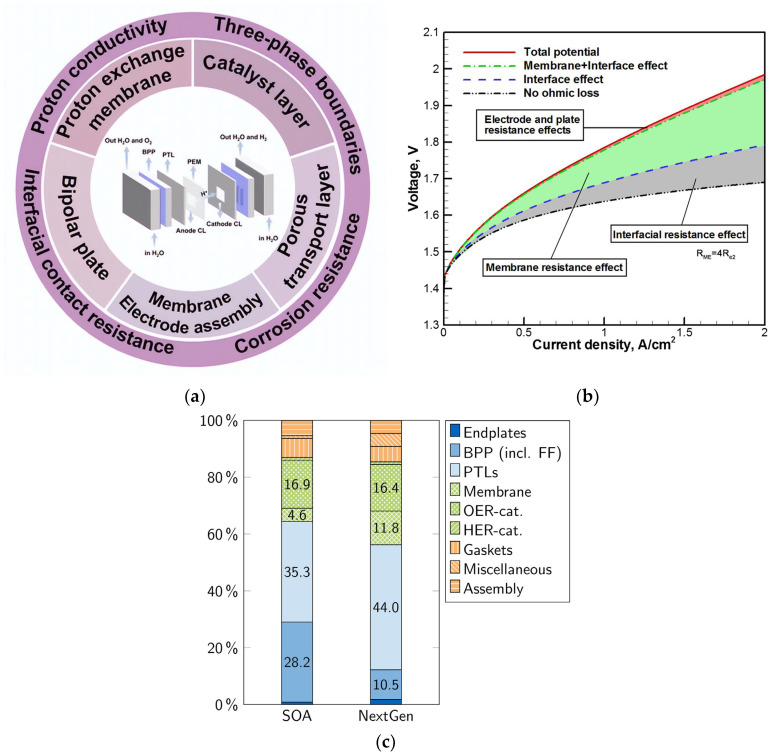
(**a**) Overall components of a PEM water electrolysis. Copyright 2023, with permission from Wiley [51]. (**b**) Influence of different component resistance in PEM water electrolysis. Copyright 2015, with permission from Elsevier [56]. (**c**) Cost analysis of PEM water electrolysis stack components of present and next-generation systems. Copyright 2023, with permission from Elsevier [81].

In PEMWE systems, the proton exchange membrane (PEM) works as a unique polymer conductor that allows protons to pass while blocking gases from a crossover. The polymer material used exhibits a high ionic conductivity. With advances in the PEM water electrolysis technology, some studies have been carried out at high current densities of 10 A cm^−2^ at 80 °C [50]. However, the biggest impediment to the commercialization of PEM water electrolysis is the high cost of the construction materials used. Therefore, it becomes critical to investigate innovative materials suitable for PEMWE. A schematic diagram of the overall components of the PEM water electrolysis is presented in Figure 4a. PEM materials include the current distributor plates, porous transport layer, catalyst-coated membrane, and bipolar plates [51]. Typically, Ti material is used for making current collector/flow field patterns as it offers strong resistance against corrosion. It is also well-suited for high voltage above 2 V, especially towards the anode side where oxidation occurs [52]. The Ti plates used are generally coated with precious metals like gold and platinum [53,54]. The following shows percentages of the costs of the stack components: MEA, 36%; PTLs, 32%; BPPs, assembly process, 8%; and stack miscellaneous, 13% [55]. The effects of different components such as electrode and plate resistance, membrane resistance, and interfacial resistance that contribute to the overall energy losses are shown in Figure 4b [56]. Cost analysis of the current generation of PEM water electrolysis reveals that PTLs and BPPs account for a significant percentage of the cell costs. Figure 4c illustrates that cost reductions can be anticipated for some components, especially BBP and catalyst loading costs, of the next-generation stack system. However, some other components such as PTLs and end plates are predicted to remain unchanged despite these reductions, which may raise their cost share in future cells.

**Figure 5:**
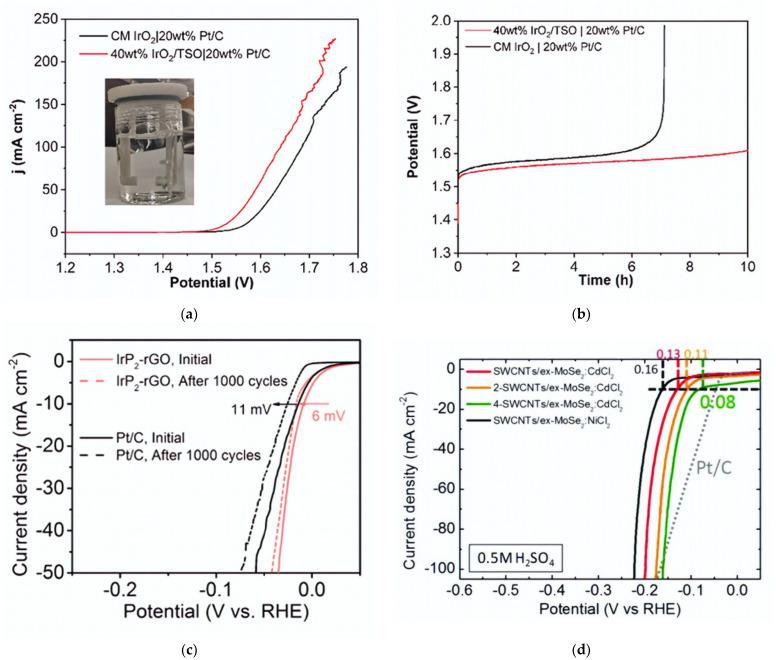
Comparison of IrO_2_ catalyst doped with and without Ti-SnO_2_: (**a**) Anode side 40 wt.%. IrO_2_-TSO and cathode side with 20 wt.% Pt/C. Copyright 2023, with permission from the American Chemical Society [57]. (**b**) Durability test at constant current 10 mAcm^−2^. Copyright 2023, with permission from the American Chemical Society [57]. (**c**) Cathode side HER with Pt/C and IrP_2_-rGO. Copyright 2020, with permission from the American Chemical Society [73]. (**d**) Single-wall carbon nanotubes/exfoliated MoSe_2_ doped with CdCl_2_. Copyright 2018, with permission from Wiley [61].

**Figure 6:**
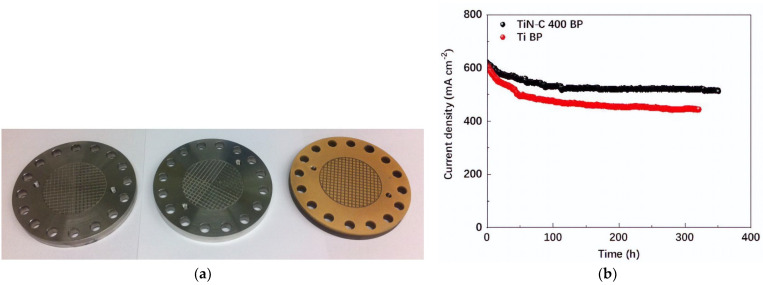
(**a**) Three different BPPs such as Ti, stainless steel, and Au-Ti. Copyright 2014, with permission from IOP Publishing [74]. (**b**) Durability test between Ti and TiN-C coating in BPP. Copyright 2023, with permission from Elsevier [75].

IrO_2_ catalyst is doped with 5% Ti-SnO_2_, which shows a larger area available for catalyst reaction [57]. As shown in Figure 5a,b, IrO_2_ doped with Ti-SnO_2_ and Pt/C (20% wt. of Pt) outperformed commercial IrO_2_. It also exhibited better durability and prolonged lifespan in the water electrolysis process compared to normal IrO_2_ catalysts. Once water enters the anode side, oxygen and protons are generated at the OER. The proton is eventually moved, where it interacts with an electron to form hydrogen at the cathode side [58]. OER refers to the process that produces oxygen on the anode, while HER refers to the process of generating hydrogen on the cathode. The HER process is the primary essential reaction required to produce hydrogen gas from electrolysis. Platinum metal groups (PGM) such as Pt, Ru, Pd, and Ir are common catalysts used in HER due to their electrochemical resilience in acidic conditions [58,59]. However, PGM have a low supply and high price, so there is significant interest in researching alternate catalyst materials [60]. Apart from the PGM, there are other alternative catalyst materials such as hybrid catalysts (i.e., Cl-MoSe_2_ and Cl-metal oxide hydr(oxy)oxide) [61], Ru dopped on Ti_3_C_2_T_x_ and Co-N-C [62,63], transition metals (Fe, Co, Zn, Cd) [64], high-entropy alloys (such as CoCrFeNiAl) [65], atomically dispersed catalysts (Ni, Co, Fe, Mo) [66,67,68], and metal-free catalysts (e.g., single-walled carbon nanotube, graphene, red phosphorous) [69,70,71,72]. Some studies have also reported that IrP_2_-rGO and single-wall carbon nanotubes/exfoliated MoSe_2_ doped with CdCl_2_ exhibit good performance in HER as shown in Figure 5c,d [61,73]. Bipolar plates with flow channels play a significant role in PEM water electrolysis. They are utilized for eliminating gases, conveying heat and electric current, and maintaining the general stability of the system [58]. Three different materials (Ti, stainless steel, and Au coated with Ti) are used as BPPs for testing corrosion resistance as shown in Figure 6a. The results of testing the corrosion resistance showed that the uncoated material stainless steel was more prone to corrosion, while the coated Au-Ti significantly lowered the interfacial resistance [74]. Thus, Au-Ti exhibits a strong corrosion resistance which in turn can enhance the electrical conductivity between the plates and other components in the cell. Another study has incorporated TiN-C and compared it with a conventional Ti BP through a test conducted at 80 °C for 300 h [75]. It showed that the corrosion resistance and long-term stability of bipolar plates in PEMWE systems could be enhanced by coating with TiN-C 400, which exhibited more positive conductivity and durability than the normal Ti BP, as shown in Figure 6b. Rojas et al. [76] have used different coated materials for BPs, such as CrN-TiN, bare stainless steel, TiN-Stainless steel 316L/Stainless steel 904L, Ti/TiN-Stainless steel 316L/Stainless steel 904L, TiN-Stainless steel 321, Ti/TiN and Ti monolayers on Stainless steel 321, and TiN monolayers. The use of a parallel plate attached to the BBP results in quicker bubble elimination [77]. Another study has used an all-in-one bipolar electrode where different components are combined to make a single bipolar electrode with an ultra-catalyst loading of 0.2 mg Pt/cm^2^, significantly lower than the 3.0 mg Pt/cm^2^ of normal CCM [78]. They used a pin-type flow channel for the effective transport of bubbles inside the channel. A BP without flow channels may significantly lower cell costs. However, it may introduce other issues such as increased pressure drop, which could inhibit the removal of bubbles within the mass transport area [42]. Another study has used the cathode side with a bipolar plate made of Ti metal using 10 parallel flow channels to improve the pathway for effective electron movement and heat management during the evolution of the hydrogen bubble. This BP can perform heat dissipation during electrochemical reactions, preventing an excessive rise in temperatures. The IrRuO_x_ catalyst of 3.0 mg/cm^2^ was coated on the anode side and 3.0 mg/cm^2^ of Pt black on the cathode side of the MEA [5]. Table 2 presents the different materials used for PEM water electrolysis owing to their tensile strength, substantial expansion, adequate flexibility, and low cost. While titanium alloys have strong corrosion resistance and aluminum has equivalent qualities, stainless steel achieves a satisfactory equilibrium in mechanical performance with easy manufacturing and low product cost. It has the potential for potent bipolar plate fabrication. However, when exposed to H_2_ gas, particularly at temperatures above 80°, Ti BPP forms hydride (TiH_2_), causing H_2_ embrittlement [79,80]. This may generate some fissures in the material, which can influence the nucleation and growth of bubbles on electrode surfaces. Depending on how unstable the gas evolution is, the bubbles that form on the electrode surfaces may react significantly. Effective measurement of the pH value can be achieved by measuring both the anode and cathode electrodes [79].

## 3. Effects of Bubbles on Polarization Losses in PEM Water Electrolysis

**Figure 7:**
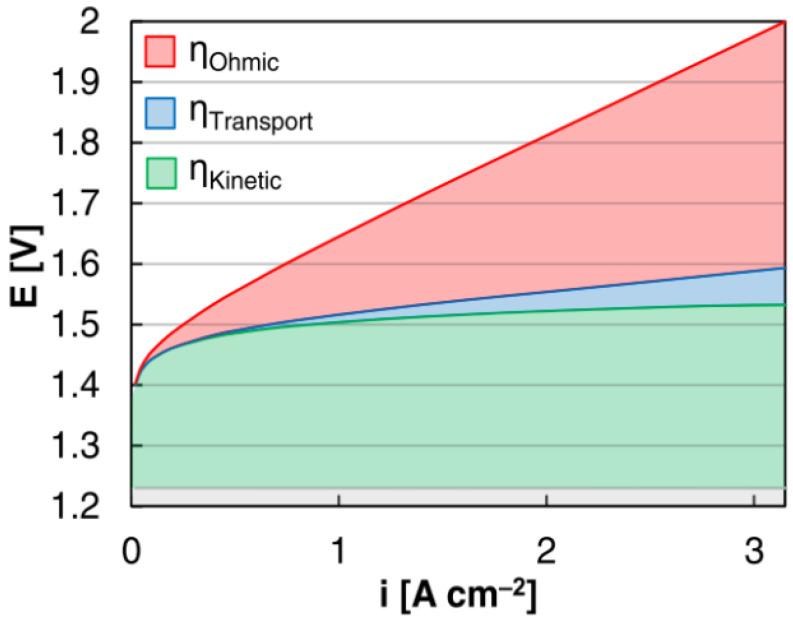
Different stages of voltage losses in water electrolysis. Copyright 2020, with permission from IOP Publishing [93].

The operating voltage at a certain current density can be used to investigate the efficiency of PEM water electrolysis. The efficiency loss in the cell can be identified by measuring the difference between the actual voltage and the equilibrium voltage for a specific current density [92]. This loss can be obtained from activation overpotential, ohmic overpotential, and mass transport losses. A polarization curve, which depicts the relationship between them, is shown in Figure 7. As depicted in the figure, the mass transport loss in PEM water electrolysis is found to be small while the activation overpotential is greater because a large amount of energy is used during the electrochemical reaction. The overpotential of a single PEM electrolyzer cell is influenced by different factors such as the activation overpotential, ohmic loss overpotential, and diffusion overpotential, which is expressed as follows [56]:

(4) $$V=V_{ocv}+V_{act}+V_{ohm}+V_{mass}$$

where *V_ocv_* is the open circuit voltage, *V_act_* is the activation overpotential which is the additional voltage desirable for activating an electrochemical reaction, *V_ohm_* is the ohmic overpotential caused by the resistance offered due to the movement of charged particles, and *V_mass_* is the diffusion overpotential due to mass transfer. Bubbles occur at the electrodes during the water-splitting process, which has a significant impact on the overall system losses [37].

It has been found that when the bubbles remain on the electrode surface, the reaction kinetics are hampered and the current flow in water is decreased, resulting in activation losses. Furthermore, if the catalyst coating on both the anode and cathode sides is exceeded in ionomer material, it might impede proton ion transit, resulting in voltage losses [93]. To comprehend these implications, HER in an acid medium can be considered where the H^+^ ion traveled from the anode to the cathode area. Charged particles (H^+^) can then change into H_2_ gas at the cathode catalyst surface and migrate away from the electrode surface, allowing the new gas bubble to form [94,95].

### 3.1. Impact of Bubbles on Activation Potential

The activation potential (*V_act_*) measures the degree of kinetic loss during electrochemical reactions [16]. Activation loss takes place on the electrode surface. It is also influenced by other factors including the number of surface-coverage bubbles [96]. With an increase in the number of bubbles on the electrode, the activation overpotential also increases to overcome the resistance offered by the bubbles. Based on the numerical study for the effect of bubble evaluation performed, low temperatures (30 °C and 60 °C) require higher activation overpotential, whereas high temperatures (90 °C) require less additional voltage energy when there is sufficient bubble coverage [97]. In many industrial processes, an excessive number of bubbles on the electrode surface might impede the proper reaction of the reactants and the product sides [98]. Bubble coverage on the electrode is also influenced by different factors such as current density and the nature of an electrode surface. Bubble coverages on electrodes need to be managed not only for activation losses but also for other losses such as ohmic losses and mass transport losses [37,99]. As a result, it is critical to determine how much of the active electrode area is covered by the surrounding bubbles, which is expressed as follows [100]:

(5) $$i_{act}=\frac{I}{\left(1-\Theta \right)A_{proj}}=\frac{i}{1-\Theta }$$

where *I* is the applied current, *A_proj_* is the projected electrode area, and Θ is the portion of the electrode surface covered by bubbles. Therefore, the activation overpotential (*V_act_*) for both the anode and the cathodic side is expressed as follows [101,102,103]:

(6) $$V_{act}=\frac{RT}{\alpha ZF}\ln\left(\frac{i}{\bar{\theta }i_{0}\left(T\right)}\right)$$

where *R* is the gas constant, *Z* is the stoichiometric number that denotes the number of electrons transferred over a specific electrochemical reaction, *α* is the charge transfer coefficient (CCC) of the anode and cathode side and $\bar{\theta }$ is the anodic and cathodic activation fraction.

### 3.2. Impact of Bubbles on Ohmic Overpotential

The additional energy required by the system to overcome the impedance produced by protons as they move over the membrane electrode is known as the ohmic overpotential. Ohmic losses can result from both electronic losses and ionic losses. Electronic losses occur when the electric current faces resistance in components such as bipolar plates, electrodes, and current collector plates [104]. Conversely, the ionic losses are linked to the resistance faced by the proton as it transfers through the membrane. The nature of the material used for fabrication and the method employed during the process are key factors that determine how much overpotential energy is required [105]. Ohm’s law is the function of total resistance to that of current density, which is expressed as [106]

(7) $$V_{ohm}=IR_{total}=I(R_{e}+R_{i})$$

where *I* is the current density and *R_e_* and *R_i_* are the resistance contributed by electronic and ionic.

Increased gas evolution can significantly increase the number of bubbles coverage on the electrode surface, which may contribute to ohmic losses [107]. The formation of bubble layers on the electrode surface may hinder direct contact between the electrode and electrolysis by blocking a portion of the active electrode surface area for gas evolution, resulting in a decrease in electrode efficiency [24]. Proper bubble management can effectively control gas evolution and enhance electrolysis. Increased gas evolution can significantly increase the number of bubbles on the electrode surface, which can lead to increased ohmic losses [107]. In PEM water electrolysis, the ability of the ionomer to conduct the proton is influenced by the presence of water. A low proton conductivity can result in decreased ion transfer across the membrane, which can increase ohmic losses within the cell [92].

### 3.3. Impact of Bubbles on Mass Transport Overpotential

This is also referred to as mass transport overpotential, which corresponds to the additional voltage energy desired to overcome the restrictions produced by the transfer of reactants and end products between electrodes in a PEM water electrolysis. The diffusion overpotential can be calculated using the Nernst equation as follows [108]:

(8) $$V_{diff}=\frac{RT}{nF}\ln\frac{C}{C_{rf}}$$

where *R* is the universal gas constant, *T* is the temperature, *C* indicates the amount of oxygen gas concentration at the electrode surface, *C_rf_* is the reference concentration, and *F* is the Faraday constant.

Water flow can impact mass transport losses either by removing bubbles in the cell or by other factors unrelated to bubble removal [41]. The impact of mass transport has been investigated in a 2D system for predicting the polarization curve. However, that study was solely bound to the theoretical Nernstian approach. It emphasized primarily how substances moved inside via diffusion [108]. Mass transfer losses occur solely in the anode region due to its complex two-phase flow dynamics. They are more pronounced when the applied current density exceeds 1 Acm^−2^ [109]. When operating PEM water electrolysis at a low current density, the main contributors to overpotential losses are sources other than mass transport overpotential. Mass transfer overpotential performance influence is often insignificant under these conditions [110].

## 4. Bubble Formation in PEMWE

**Figure 8:**
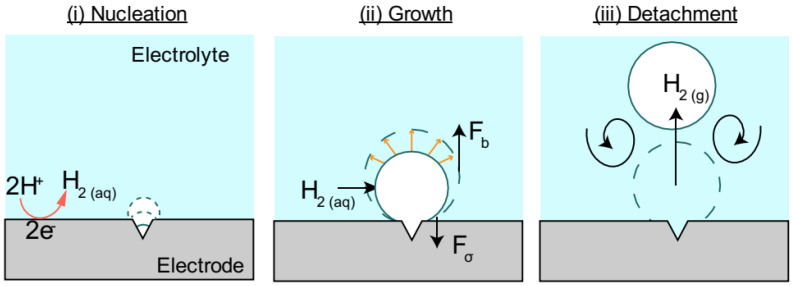
Different stages of bubble formation. Copyright 2020, with permission from Elsevier [37].

The formation of gas bubbles at the anode and cathode surfaces can affect the production of hydrogen gas in PEM water electrolysis. The growth of oxygen gas bubbles can be controlled by changing several parameters such as current densities and temperatures [17]. When two distinct surfaces, i.e., hydrophilic and hydrophobic surfaces, were employed for a bubble detachment study, the results demonstrated that the influence of the wettability surface was less important at lower current densities [111]. It is vital to understand how different components influence voltage in a water electrolysis system and the movement of bubbles. How bubbles develop and migrate and how they disturb different parts of the cell are still not well understood. Previous reports have suggested that excess bubble formation can delay the detachment of bubbles and lead to insufficient bubble growth in the cell. As a result, considerable mass transfer loss, reduced catalyst utilization, and unpredictable cell performance can occur [92]. Therefore, there is a substantial need for developing practical and well-considered methods for handling and controlling bubbles. For instance, Yaun et al. [92] have developed a novel electrode to improve bubble management by utilizing an anode catalyst that is 24 times more effective than the standard design. This PEMWE is more adaptable than the standard technique in terms of energy efficiency, oxidation, lightweight design, and output rate [35]. Nonetheless, practical manufacturing of hydrogen/oxygen gas has encountered some significant challenges, including high component costs, GDL lifespan, and its pricey coated catalyst layer [112]. Enhancing bubble evolution and transport are two primary aspects of bubble management in water electrolysis [113]. In the first procedure, bubbles that emerge from the catalyst layer (CL) must be removed as quickly as possible [92]. Several studies have shown that a porous transport layer (PTL) plays a crucial role in removing bubbles. By altering its structure, it can enable bubbles to flow more freely and decrease the efficiency loss induced by bubbles [49]. For example, drilling holes in the PTL can save up to 76.7% of the efficiency loss [114]. Another study has developed a novel catalyst design that allows bubbles to move more quickly and effectively by coating the layer at the edge of Ti foil [115]. For enhancing bubble transport, the process can be carried out by generating more nucleation sites where smaller bubbles can develop when they detach. By allowing bubbles to detach more, the oxygen in water will be reduced, which will improve the system [92,99]. Studying characteristics such as the nucleation, growth, and detachment of bubbles under different operating conditions is necessary to comprehend the dynamics of bubbles in the flow channel and LGDL [35]. Figure 8 illustrates the evolution of the bubble stages in electrodes. In PEM water electrolysis, hydrogen bubbles produced are smaller than oxygen bubbles due to their difference in stoichiometry.

Ultrasound has a notable impact on the optimization of bubble evolution in water electrolysis. Ultrasound enhances nucleation, and it also influences bubble growth and allows bubble detachment from electrode surfaces via cavitation. Under the influence of an ultrasonic wave, the bubbles tend to merge into larger bubbles, along with an increase in bubble velocity on the electrode surface [116]. They have also noticed a decrease in critical bubble diameter and residence time under sonication. When the ultrasonic wave is applied, cavitation bubbles form because of rapid changes in pressure within the liquid. When ultrasound cavitation is applied in the pure water splitting process, the production of hydrogen and oxygen doubles. This improvement is due to the rapid release of the oxygen bubble induced by the ultrasonic effect. As a result, it minimizes the chance of dissolved oxygen and hydrogen merging, resulting in a more effective catalytic process [117]. During cavitation, rapid pressure changes induce bubbles in a liquid to grow and collapse quickly, especially when the negative pressure falls below the saturated vapor pressure [118]. During this stage, the bubbles promptly nucleate and grow. When bubbles enter a positive pressure zone, they collapse abruptly and explosively due to the pressure exerted by the surrounding liquid. This sudden increase in pressure has a variety of repercussions, including mechanical stress, increased turbulence, and alterations in the electrochemical environment, especially when it collides with nearby electrode surfaces. The cavitation of bubbles is influenced by the frequency of the ultrasound waves, and higher frequencies usually result in a stronger cavitation impact. The sound waves cause pressure to fluctuate. When these bubbles collapse, it creates a strong localized force and sound waves and this leads to intense mixing, which produces a faster reaction rate and higher efficiency. Ultrasonic waves offer an advantage in photocatalytic water splitting via piezoelectric action due to their high transmission frequency and deep penetration in water [117]. While this discussion on ultrasound’s impact on bubbles was based on general water electrolysis, it is important to highlight that the same concept may apply to PEM water electrolysis.

### 4.1. Nucleation of Bubbles

Nucleation is the process in which the first gas bubbles appear on the electrode surface. It happens when there is a dissolved gas buildup at the electrode surface [119]. If the dissolved gas concentration exceeds its saturation concentration during electrolysis, the gas will subsequently cause bubbles. How bubbles form, grow, and separate from the electrode surfaces affects the volume of gas released from the surrounding surface, which is crucial for determining the rate of bubble formation [120]. In PEM water electrolysis, the driving force for bubble nucleation depends on the degree of supersaturation of dissolved oxygen at the anode and hydrogen at the cathode [121,122]. Hydrogen and oxygen molecules are generated by electrochemical processes on the electrode surface and supersaturated to produce bubbles [123]. Bubbles become detached from the surface once they reach a critical size diameter. The critical bubble size for oversaturation within pores can be calculated using the classical nucleation theory as follows [25].

(9) $$R_{c}=\frac{2\gamma }{P_{g}-P_{l}}$$

(10) $$P_{g}=S\cdot P_{l}$$

where *R_c_* is the minimal size at which the bubble nucleates, *S* is the supersaturation of dissolved oxygen in the PTL pore, *γ* is the water surface tension, *P_g_* is the pressure inside the bubble, and *P_l_* is the surrounding water pressure.

Two processes are involved in the nucleation of bubbles: a homogeneous nucleation process, in which bubbles nucleate within the liquid without any solid surfaces, and a heterogeneous nucleation process, in which the formation of bubbles is facilitated by the presence of a solid surface [124,125]. Bubble nucleation is significantly influenced by non-wettable surfaces [37]. In the water electrolysis process, the generation of bubbles occurs at the interface between the catalyst layer (CL) and the porous liquid gas diffusion layer (LGDL) [18]. Bubbles then combine with their neighbors to form a cluster structure, thereby increasing their volume and surface energy [25]. Once these cluster bubbles reach a critical size, they disengage from the nucleation sites, resulting in the formation of new bubbles [126]. Another study has found that increasing porosities and decreasing pore size can lead to higher bubble nucleation at the triple-phase boundary [127]. The addition of a hydrophobic layer between the electrocatalyst and the PTL anode-side LGDL increases the contact angle, resulting in more bubbles on the surface, particularly at low current densities [128]. Efficient management of pressure is essential for controlling the bubble behavior inside the system. The bubble behavior is influenced by current density, temperature, and the liquid flow rate. In contrast, current density and temperature have considerable impacts on bubble nucleation sites, growth, and the overall number of bubbles [40]. Increasing current density accelerates the initiation of bubbles (bubble nucleation), resulting in quicker bubble formation on the anode catalyst layer [35].

Bubble nucleation takes place within a frequency range of 10 to 50 Hz. Nucleated bubbles will detach from the CL while the shape of bubble growth resembles a semispherical cap inside the PTL pore [25]. Previous studies have found that ideal areas for bubble nucleation can result in a heterogeneous crack on the catalyst surface as they are less prone to water movement or external forces [127,129,130]. The size of the catalyst crack surface can influence how the initial bubble forms and grows. In PEM water electrolysis, bubble density nucleation is analogous to a nucleation site in a boiling pool, where vapor bubbles develop on a heated surface in contact with water [25]. Different techniques are available for isolating the nucleation of bubbles and examining their impact on the electrode that evolves gas. Nucleation of bubbles usually emerges when there is an active OER. However, it is crucial to note that bubbles may not always appear at the precise location where the reaction occurs [92]. Figure 8 illustrates how the nucleation of bubbles results in water electrolysis. Nucleation is due to a constant increase in dissolved gas beneath the catalyst surface (*C_gas_*). The water electrolyzer action introduces gas molecules into the water phase, increasing *C_gas_* above its saturation concentration, *C_sat_*. The equilibrium concentration of saturation gas is proportional to the partial pressure of the gas (Henry’s Law) as shown below [37,92].

(11) $$C_{sat}=PK_{H}(T)$$

Henry’s solubility constant *K_H_* decreases with temperature for each pair of liquid–gas molecules [131,132]. The supersaturation of gas (*ζ*) results when the dissolved gas is large enough, which is expressed as follows:

(12) $$\zeta =\frac{C_{gas}-C_{sat}}{C_{sat}}$$

**Figure 9:**
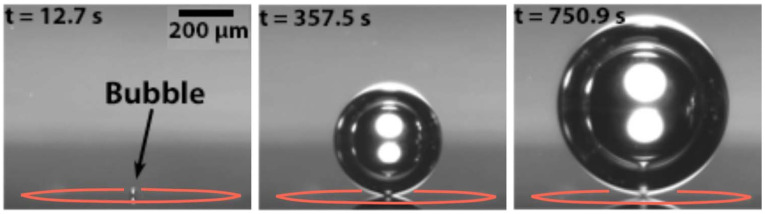
Evolution of bubble from nucleation and growth of hydrogen bubble on SiO_2_ substrate. Copyright 2019, with permission from IOP Publishing [133].

A novel ring-shaped electrode has been used to study the formation of bubbles. However, it was discovered that the catalyst-free center of the ring was where the bubbles formed, which is shown in Figure 9 [133]. 

The bubble nucleation starts after a few seconds of the reaction. This occurs because the dissolved gas congregates in a hydrophobic microgravity area. The liquid is supersaturated when *ζ* > 0 (*C_gas_* > *C_sat_*) and unsaturated if *ζ* < 0 (*C_gas_* < *C_sat_*) [134]. To avoid bubble formation near the catalyst surface, the bubble generation and gas evolution reactions must be separated. This can help prevent the overpotential effect of gas evolution in the bubble formation method [135,136].

### 4.2. Growth of Bubbles

**Figure 10:**
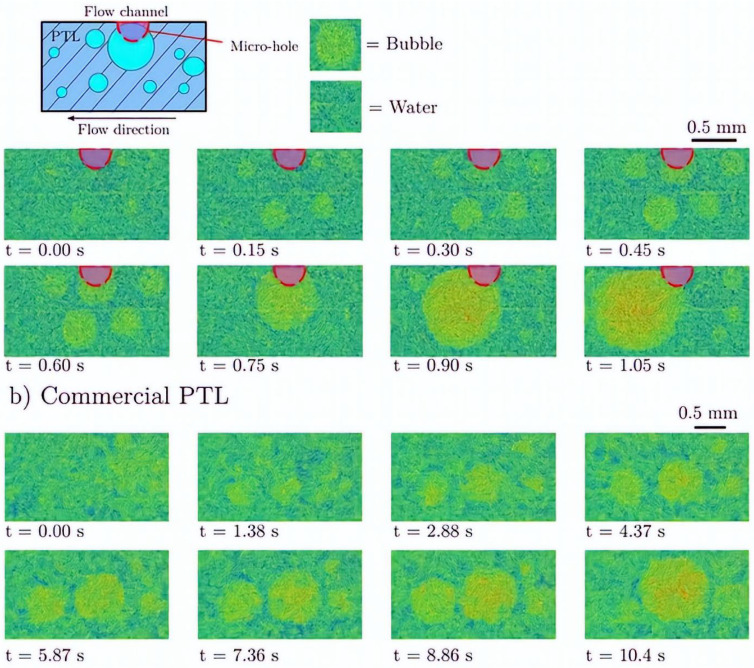
Bubble growth in through-pore PTL and commercial pore PTL at different times. Copyright 2020, with permission from American Chemical Society [144].

After the nucleation stage, the subsequent phase involves bubble growth which depends on various properties such as surface tension, viscosity, inertia, and turbulence [120]. In electrolysis, the growth of bubbles involves three phases, each of which is governed by a distinct force. The initial growth phase is regulated by the liquid inertia that surrounds the bubble. At this point, the radius of the bubble increases quickly with time [137]. The second stage happens when the dissolved gas surrounding bubbles in the liquid becomes supersaturated. Based on how quickly the gas can permeate from the bulk liquid, the bubble growth rate is constrained. The bubble radius increases with the square root of time [134]. In the third stage, the electrode produces more gas than it can dissipate into a bubble. Bubble growth frequency decreases because of the electrochemical process [36,138]. In PEM water electrolysis, bubble growth is governed by the difference in concentration between the dissolved gas molecules and the solubility of a gas in water [92]. The gas solubility in water increases with temperature. As a result, bubble growth accelerates significantly with a high temperature. The bubble growth rate is dependent on different factors including geometrical design, pressure, temperature, current densities, and surfactants [17,139]. Bubble growth in water electrolysis is shown in Figure 8. Some bubbles grow continuously even after bubbles have detached from the surface, indicating that as long as the surrounding liquid remains supersaturated, the bubble size will continue to increase [40,140]. However, the inability to observe inside the microstructure of opaque cells has resulted in a relatively limited understanding of bubble growth [141]. Numerous studies have postulated a two-phase bubble transport in a porous transport layer (PTL). A conventional PTL degrades faster than a modified PTL which exhibits higher durability and contact mass transport [142,143]. A PTL with a patterned structure and perforated pores can significantly improve bubble management in an electrolyzer [144,145]. A study has compared the growth of bubbles and detachment of bubbles with a through-pore PTL and a normal pore structure PTL and found that the through-pore PTL has higher gas flow [144]. It has also been found that the through-pore-type PTL has better water movement by enhancing the mass transport which is critical for system efficiency. However, some pores are inactive in a through-pore PTL, which results in an ineffective in-plane water movement [146]. Bubble growth took only 0.3 s with a through-pore PTL, whereas it took 2.88 s with the regular-pore PTL. This suggests that the through-pore PTL is more effective for higher gas removal, as shown in Figure 10. Furthermore, bubbles emerging from the through-pore PTL can continue their growth until they come together with surrounding bubbles, as seen in the through-pore PTL at t = 0.75 s.

### 4.3. Detachment of Bubbles

**Figure 11:**
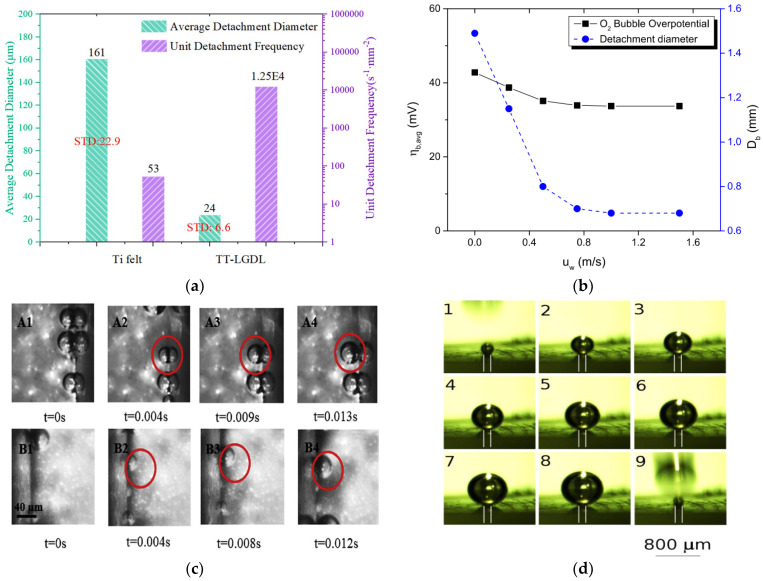
(**a**) Comparison of bubble detachment between commercial Ti and hybrid coating (Ti-LGDL). Copyright 2021, with permission from Elsevier [30]. (**b**) Effects of water flow rate velocity on bubble overpotential and the bubble detachment. Copyright 2017, with permission from Elsevier [25]. (**c**) Detachment of O_2_ and H_2_ bubbles from a Pt electrode. Copyright 2019, with permission from Elsevier [40]. (**d**) Different stages of H_2_ bubble growth. Copyright 2014, with permission from the American Chemical Society [135].

Bubble detachment occurs when the upward force of buoyancy dominates the downward force of surface tension. Two main forces that can affect bubble departure in a pool of liquid are the force of buoyancy and the force of surface tension [147]. Bubble detachment diameter (*D_d_*) can be calculated by correlating the surface tension force (*γ*) with the buoyancy force, which is expressed as [125,148].

(13) $$D_{d}\pi \gamma \;\sin^{2}\theta =\Delta \rho gf\left(\theta \right)D_{d}^{3}$$

where *ρ* is the liquid density, *θ* the contact angle between a bubble and the surface, Δ*ρ* is the density difference between water and gas bubble, and *g* is the acceleration due to gravity experienced by the bubble. The frequency *f* based on the different bubble detachment diameters has been presented previously [149]. Although research on active catalysts has been significantly advanced over the years, very few studies have been conducted on electrocatalyst activities to effectively design suitable bubble detachment from the electrode surface [150]. For quick removal of bubbles from the surface, it is essential to lower the adhesion between the surface and bubbles [151]. In the water electrolysis process, there are various methods to detach the bubble from the electrode surface by using magnets, sound waves, mechanical frequency, and a supergravity field. The study also suggested that effective bubble detachment requires changing the liquid composition and system design. These techniques can facilitate the movement of forces in a liquid, which can accelerate the movement of bubbles on the surface [150]. Generally, more homogeneous and smaller bubbles can be removed from the cell faster, allowing more water to pass through [30]. They studied the bubble transport mechanism using a PTL as titanium felt with PTL and thin titanium with LGDL and compared three different indexes (i.e., bubble average detachment diameter (ADD), standard deviation diameter (SDD), and detachment frequency (DF)). Figure 11a shows the average bubble and detachment frequency for both PTLs. The ADD was 161 µm for Ti-felt and 24 µm for TT in the LGDL. The bubble formed by TT in the presence of the LGDL was found to be more uniform and smaller. Furthermore, the DF for TT in the LGDL was higher than that for Ti-felt. The growth of bubbles and stability on flat electrodes and the PTL flow channel interface can be changed with advanced imaging technologies [25]. Bubble detachment occurs due to two primary factors: (1) bubble nucleation near the anode catalyst layer caused by increased localized oxygen supersaturation in that region, resulting in the formation of fresh bubbles; and (2) balancing forces such as pressure and buoyancy. When these forces reach their critical point, the bubble detaches from its origin [25]. Figure 11b shows the effects of liquid velocity on bubble overpotential and bubble detachment. Liquid velocity plays a significant role in bubble management. When the flow velocity is low, the bubble tends to merge and form a large slug, which requires more time to detach. Similarly, when the flow velocity is increased, it is more difficult for smaller bubbles to clutch together due to the higher pressure difference and shear stress at the bubble surface. The detachment of hydrogen bubbles on the cathode side is always lower than the detachment of oxygen bubbles on the anode side [40].

Once the bubble detaches from the cell, the concentration of dissolved gas starts to increase in water, and when it reaches a particular level, the nucleation process starts, which initiates the bubble evolution cycle [36,152]. Bubble detachment of H_2_ and O_2_ at constant current density and flow velocity with time is depicted in Figure 11c. The bubble detachment study has been conducted using neutron and X-ray imaging methods. It has shown that initially, the bubble detaches very rapidly, and it slows down until it reaches a critical size [86]. Figure 11d presents the different stages of bubble growth on microelectrodes. Here, bubbles are generated and detached more consistently and progressively instead of detaching all the bubbles at once [135]. Garcia-Navarro et al. [153] used MATLAB code and studied O_2_ bubble detachment and explained that the bubble detachment remains constant regardless of the change in water flow rate. However, some other researchers have reported that an increase in water flow velocity leads to quicker detachment of smaller bubbles [154,155]. At constant operating potential, the size of bubbles decreases with an increase in the water flow rate. This phenomenon can be explained when water flow increases when water flow increases it creates less space (void fraction) available for gas bubbles. As the bubble rises, it displaces the water, causing transverse motion. Larger bubbles at a certain range of size contributed to water displacement and thus swept away other bubbles on the electrode surface. In the present review study, the intricate discussions on bubble nucleation, growth, detachment, and the effects of catalysts are presented mostly in the context of general water electrolysis. However, the underlying concepts of bubble dynamics and its applications may also be applied similarly in the context of PEM water electrolysis. This broad fundamental study of bubbles can result more specialized area of PEM water electrolysis specialized area of PEM water electrolysis.

## 5. Bubble Dynamics in Different Components of PEM Water Electrolysis

When a voltage is supplied during the water electrolysis process, then O_2_ and H_2_ bubbles are produced on the anode and cathode sides, respectively. The movement of bubbles is influenced by factors including buoyancy, surface tension, and drag force. The O_2_ bubbles are formed on the catalyst surface and start to grow until they reach the critical size and get detached away in the flow channel through the PTL. In most studies, water is supplied toward the anode. However, in some cases, water is also passed via the cathode-side channel to prevent the degradation of the membrane [29,156,157]. The following section discusses bubble dynamics in PEMWE with different components.

### 5.1. Bubble Dynamics in Flow Channels

**Figure 12:**
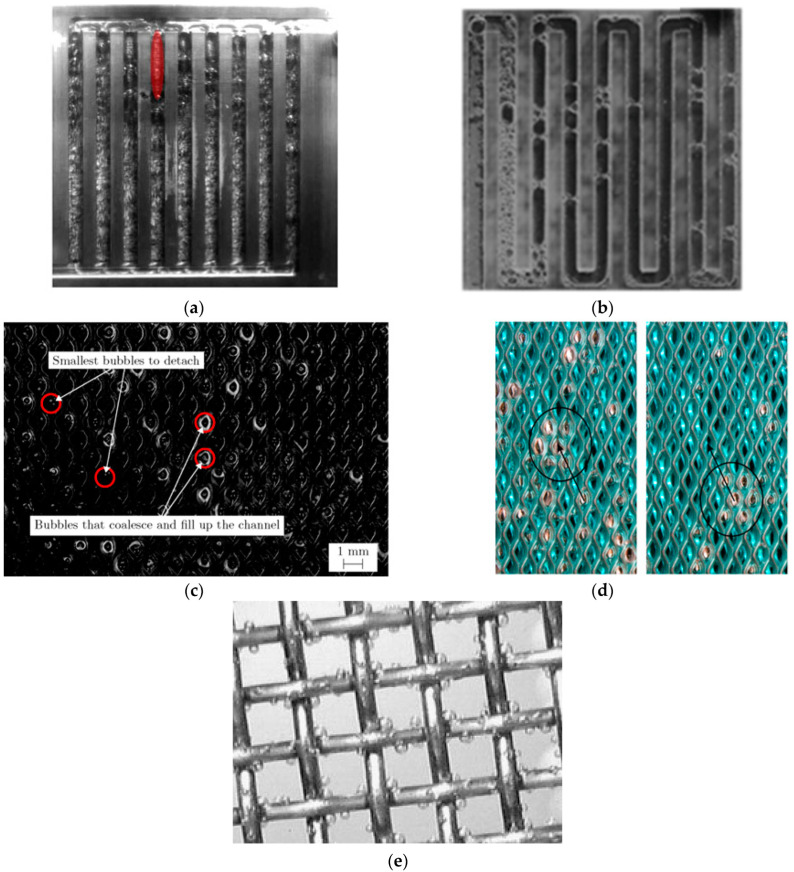
Bubble motion in different flow channels. (**a**) Parallel-type channel. Copyright 2020, with permission from Frontiers [24]. (**b**) Serpentine flow channel. Copyright 2018, with permission from Elsevier [38]. (**c**) Mesh-type channel. Copyright 2019, with permission from Elsevier [153]. (**d**) Expanded metal mesh. Copyright 2018, with permission from Elsevier [161]. (**e**) Stainless steel mesh. Copyright 2010, with permission from Elsevier [162].

The flow channel is an important structure used for designing the PEM water electrolysis. In PEM water electrolysis, various flow channels have been used for study, including serpentine, parallel, pin-type, interdigitated, mesh-type, and cascade channels [14,22,38,83]. In comparison with serpentine flow, the parallel field performs better at low pressure drop with constant flow velocity and lesser turbulence, which can increase corrosion resistance [158]. Polarization curves for various channels, including single and dual serpentine flow (SF) and parallel flow (PF) fields, have shown that more parallel channels can lead to more effective system performance [29,159]. A dual serpentine flow field is advantageous with respect to pressure drop, temperature, and current density distribution because it allows more reactants to penetrate porous layers and increase system reaction [160]. It has been found that a serpentine channel with a longer flow field produces elongated gas bubbles that can block the flow channel [38]. That research showed that, when SF and PF were compared at the same water flow, PF performed better than SF, especially at higher current densities. In the case of the SF, an annular regime was observed at high current densities. This caused the gas bubble to occupy the entire channel length, resulting in water obstruction across the LGDL and minimizing cell performance. O_2_ deposition in the channel may increase pressure drop and impede the system’s nonuniform temperature and current flow [38]. They also mentioned that while designing the flow field, significant attention must be considered for pressure drop management. The circuit board was printed, and the bubble flow was observed to investigate the current density along the system [154]. Those studies showed that the removal of gas bubbles from the electrode surface and the movement of water flow were significantly influenced by the presence of larger bubbles. This operation from smaller bubbles to larger bubbles enhanced mass transport results due to an increase in uniform current distribution across the channel. To maintain the two-phase flow as a bubbly flow, an ideal flow rate is required to enhance mass transfer and minimize overvoltage concentration [29]. Ojong et al. [42] stated that when only a BPP is used without a flow channel, pressure drop increases and the bubble formed tends to deposit throughout the PTL surface. This bubble accumulation had an adverse effect on mass transfer within the cell. Bubble motion inside the parallel channel gets restricted at a high current density, as shown in Figure 12a. Stagnant bubbles covered almost the whole channel length and made it more difficult to remove the gas [24]. Deposition of O_2_ gas bubble in a serpentine channel is more severe than in a parallel channel due to the formation of a long slug, as shown in Figure 12b [38]. The long slug flow caused a significant amount of gas bubbles to build along the channel, which considerably slowed down the movement of water and degraded system performance [40]. Similarly, Figure 12c depicts a mesh channel with two distinct types of bubbles involved: small bubbles and large bubbles. Small bubbles with a size of 30 mm detached quickly from the surface. However, a larger bubble remained attached to the adjoining bubble and eventually obstructed the PTL.

One study indicated that cascaded flow channels used on the anode side performed better than serpentine and parallel channels due to the low deposition of bubbles across the field [83]. Figure 12d depicts a zigzag flow pattern of bubbles on the expanded metal mesh. Lafmejani et al. [161] studied both single-phase and two-phase flow by injecting blue ink along the water flow and observing how it behaves in the mesh channel. The presence of the bubble along a vertical path was shown to be favorable for liquid flow. An interdigital field channel analysis of single- and two-phase flow models was performed to understand the influence of gas bubbles on the geometry structure of the anode [163,164]. It showed that unequal flow and temperature distribution in the cell was due to the equal land width of the flow field and the presence of a gas bubble at the exit phase. Maier et al. [85] used a non-invasive technique termed acoustic emission for tracking the movement of bubbles in the flow channel and this allowed them to record system changes such as the shifting of tiny bubbles to larger bubbles and changes in bubble shape in the cell. A square-shaped pin-type channel showed a consistent distribution of temperature and current, resulting in effective elimination of gas bubbles [165].

Zhang et al. [162] found that the impact of H_2_ bubbles on stainless steel (SS) mesh is influenced by the current density, mesh diameter, and pore size. They also found that the SS mesh performed better than the expanded mesh as a catalyst for hydrogen evolution, which is shown in Figure 12e.

### 5.2. Bubble Dynamics in a Catalyst Layer

**Figure 13:**
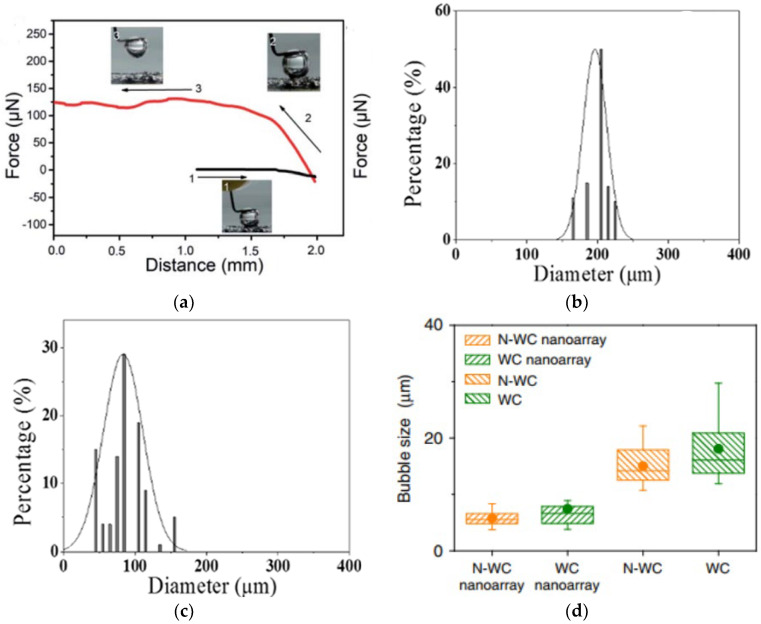
(**a**) CoMnP/Ni_2_P/NF measurement of bubble adhesive force. Copyright 2021, with permission from the Royal Society of Chemistry [178]. (**b**) BSD of FG-WS_2_. Copyright 2017, with permission from the American Chemical Society [188]. (**c**) BSD of VGNHs-WS_2_. Copyright 2017, with permission from the American Chemical Society [188]. (**d**) BSD on various nanoarrays. Copyright 2018, with permission from Springer Nature [191].

Metals such as Pt are commonly coated on the cathode side of the catalyst for examining the hydrogen evolution reaction (HER), whereas IrO_x_ is loaded on the anode-side region of the catalyst for studying the oxygen evolution reaction (OER) for coating on the membrane (CCM) [84]. However, these metals are not cost-friendly when they are used for upscaling. In addition, the use of Pt metal can be poisonous when chemicals such as sulfide (commonly found in wastewater) are used [166,167]. Nonmetal catalysts such as metal sulfide, metal carbides, and metal phosphides have been used as HER catalysts in acidic conditions [168,169,170,171]. However, these nonmetals have numerous downsides, such as consuming higher voltage energy and exhibiting weaker stability when they are subjected to higher current densities [172,173,174,175,176]. Contrary to conventional Pt and other nonmetal catalysts, a Fe-N-C catalyst has been designed for HER, showing high onset [177]. Hybrid catalysts such as CoMnP/Ni_2_P/NF showed significant activity for HER with low overpotentials in both acidic and alkaline environments [178]. As indicated in Figure 13a, this CoMnP/Ni_2_P/NF exhibits superaerophobic behavior when it is studied underwater with a high contact angle of 158° and a negligible adhesive force between the bubble and the electrode surface. During the operation of electrolysis, some cathode and anode surfaces are covered by hydrogen and oxygen gas bubbles. The diameter of these bubbles can be measured by taking two elements into account: liquid surface tension and pressure difference at the meniscus. Relationships between liquid properties, pressure difference, and bubble size can be presented as follows [179]:

(14) $$r_{b}=\frac{4T}{P_{i}-P_{0}}$$

where *P_i_* is the pressure inside the gas bubble, and *P*_0_ is the external pressure which is influenced by the height and the density of a liquid.

The performance and durability of the anode-side catalyst and anode plate are important to consider especially when it operates at a higher voltage to produce O_2_ on the anode [180]. Simply focusing on the OER may not be sufficient to prevent the catalyst from corrosion resistance [181]. They also suggested having a thorough knowledge based on different surfaces against catalyst erosion. This further understanding is essential for developing the catalyst, which not only can exhibit its life expectancy but also may resist corrosion, preventing the catalyst from leaching over time. The bubble interaction at high voltage can result in the leaching of coated catalyst material. Catalyst leaching studies were carried out for different catalyst loading, and particularly on the anode side with lower catalyst loading lower catalyst loading (0.34 mg cm^−2^) led to higher degradation rates compared to higher loading (1.27 mg cm^−2^) [182]. They stated that catalyst loading has a substantial influence on the degradation rate. If the catalyst loading is insufficient, it may fail to resist the elevated heat and ultimately it could break the catalyst material causing catalyst leaching. Compared to an MEA with low catalyst loading, an optimal loading lifespan is three times higher, which can decrease catalyst leaching and increase catalyst efficiency [183]. This nonlinear mechanical stress results specifically due to fluctuating energy supply, which in turn can cause nonuniform bubbles in electrodes that may affect the electrochemical reaction in the system [184].

**Figure 14:**
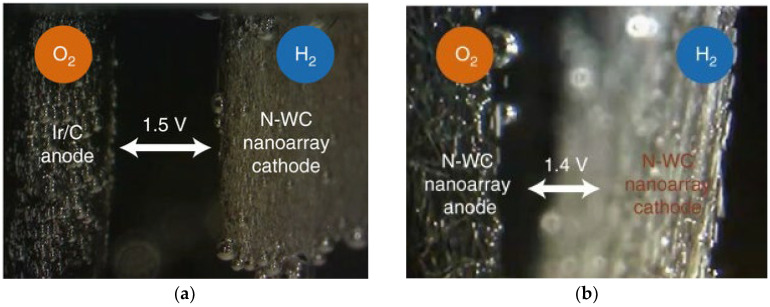

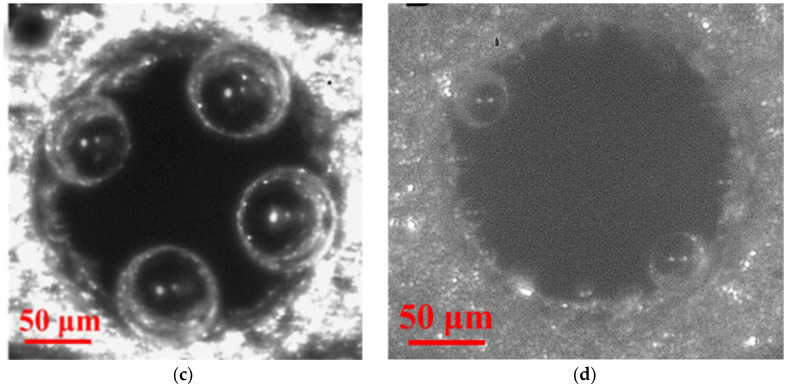
Captured bubble size images. (**a**) Ir-C coated on the anode and N-WC nanoarray on the cathode side at 1.5 V. Copyright 2018, with permission from Springer Nature [191]. (**b**) Coated N-WC nanoarray on both regions. Copyright 2018, with permission from Springer Nature [191]. (**c**) Dense Ir CCLGDL. Copyright 2023, with permission from the American Chemical Society [192]. (**d**) Honeycomb Ir CCLGDL formation of bubble dynamics in OER CCM side. Copyright 2023, with permission from the American Chemical Society [192].

Bubbles formed in the CL must exit the system via the porous transport layer (PTL) and the flow field channel [185]. Controlling the ideal catalyst loading thickness is essential for ensuring free water flow within the layer [92]. The cracks in the CL during the reaction process may lead to a negative impact on bubble management. These cracks may cause irregular and uncontrolled bubble nucleation, disrupting efficient gas transfer and system performance [186,187]. In CL, another type of surface structure known as superaerophobic surface structure serves to control gas bubbles on the OER and HER sides. This superaerophobic structure resembles an array and inhibits the bubble from adhering to the CL for an extended period [178]. For quick removal of H_2_ gas bubbles from the electrode surface, hybrid catalysts called FG-WS_2_ and VGNHs-WS_2_ have been employed and researchers measured their bubble size distributions (BDS), which are presented in Figure 13b,c [188]. The VGNHs-WS_2_ hybrid catalyst produced smaller and more uniform bubbles than the FG-WS_2_ hybrid catalyst, which was attributed to the nanorough surface of the VGNHs. As a result, the H_2_ gas bubble in the electrode can escape from the HER area faster. The Pt nanoarray shape that resembled pine showed a higher contact angle. As a result, the H_2_ bubble detached quickly from the electrode surface [189]. When two hybrid coated catalysts were used, namely, MoS_2_ flat film and MoS_2_ nanoplatelets array, the flat film showed higher adhesive force, resulting in higher bubble attachment [190]. The MoS_2_ nanoplatelets array exhibited significant bubble management with a higher bubble contact angle and thus detached bubbles faster on the surface. Han et al. [191] employed a hybrid catalyst to analyze the HER reaction side by using an N-WC nanoarray and flat N-WC. When the bubble contact angle increased from 148° (flat) to 163° (N-WC nanoarray), the N-WC nanoarray demonstrated improved bubble management with respect to the flat N-WC and detached bubble size decreased from 15 µm to 5 µm. Figure 13d exhibits the BSDs of two distinct topologies, nanoarray and non-array electrodes. The nanoarray structure produced a smaller bubble, a larger contact angle, and a lower adhesive force. The researchers examined two distinct catalysts, and in the first set, they coated Ir-C on the anode and nitrogen-tungsten carbide (N-WC) at 1.5 V. In the other set, they coated single non-noble metal catalyst N-WC on both the anode side and the cathode side at 1.4 V, as shown in Figure 14a,b. They found that using N-WC non-noble metal as a bifunctional catalyst on both the anode and cathode sides could increase the yield of the water-splitting process at a low voltage. Thus, maintaining efficient electrolysis relies significantly on managing bubbles on the HER and OER reaction sides of the CL.

On the anode, two different cost-effective catalyst coatings were performed using honeycomb Ir and dense Ir. Their comparison showed that honeycomb Ir has better bubble management due to the interconnected structures which enabled fast bubble discharge and effective water diffusion [192]. Figure 14c,d depict the bubble dynamics using a dense Ir-catalyst-coated liquid gas diffusion layer (CCLGDL) and the honeycomb (HC)-coated membrane surface. At a current density of 200 mA/cm^2^, the honeycomb catalyst layer outperformed the dense layer in terms of bubble nucleation and detachment times. The formation of gas bubbles in the CL alone was insufficient to optimize cell performance. However, how these bubbles are circulated within the cell plays a significant role in determining the overall voltage loss in the cell. This voltage is lost due to an accumulation of O_2_ bubbles on the anode side [193]. The O_2_ bubble generated on the CL takes more time to travel through the PTL when the applied current density is high, and the bubble transport is low. As a result, gas removal becomes very slow, and more bubbles build on the anode region [126,194]. Furthermore, the presence of the ionomer in the CL determined cell performance. The higher the ionomer in the CL, the more mass transfer losses, resulting in inefficient O_2_ bubble transfer [195]. This obstruction impedes efficient bubble reduction and degrades cell efficiency [93].

### 5.3. Bubble Dynamics in Porous Transport Layers

**Figure 15:**
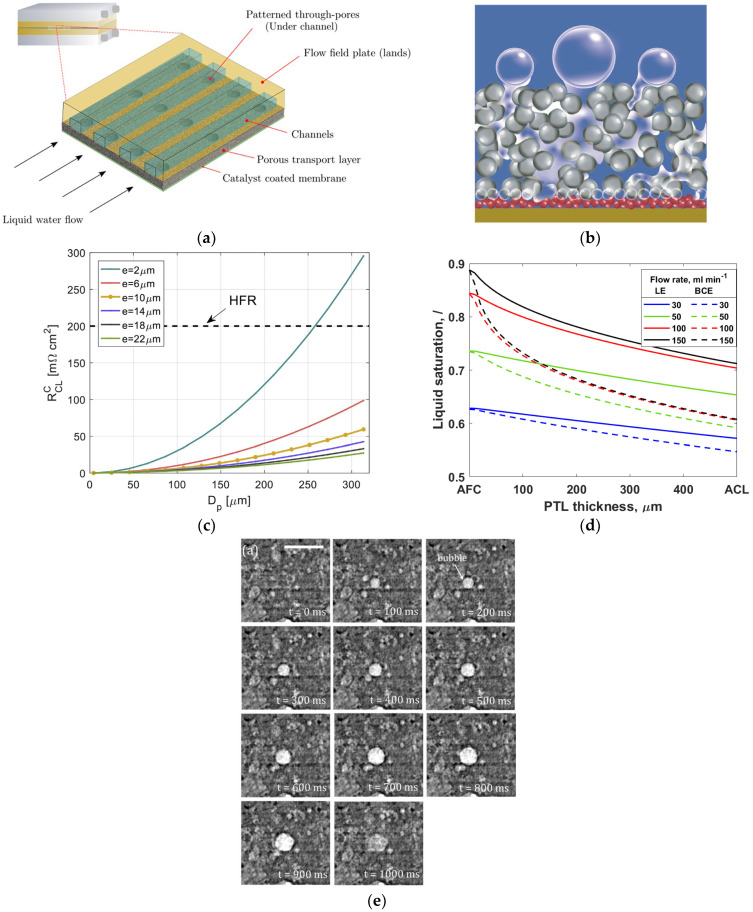
(**a**) Bubble transport patterned through-pores in PTL. Copyright 2020, with permission from the American Chemical Society [144]. (**b**) Movement of gas bubble through PTL. Copyright 2023, with permission from Elsevier [141]. (**c**) Variation in pore size on constriction resistance based on the different CL thickness. Copyright 2020, with permission from Elsevier [201]. (**d**) Influence of inlet water on PTL liquid saturation and thickness. Copyright 2022, with permission from MDPI [202]. (**e**) Formation and detachment of O_2_ bubble at different times. Copyright 2018, with permission from Elsevier [203].

A porous transport layer (PTL), also known as a liquid gas diffusion layer (LGDL), can be utilized on the anode side with a gas diffusion layer (GDL) on the cathode side. It aids in the stability of several components such as membrane, collecting current, and counter flow of gas and water [196]. The PTL with a porosity between 30% and 50% performs well for gas bubbles to navigate faster and regular flow of intake water [197,198]. If larger pores are present in the PTL interface, they can cause insufficient contact between catalyst nanoparticles and the PTL material [199]. Recently, a novel sponge-like material with small holes known as through-pores has been developed to easily move water and gas bubbles by avoiding the longer route in the PTL, as presented in Figure 15a [144]. After examining the behavior of gas bubbles in the PTL flow field using an X-ray imaging approach, it was found that bubble growth and detachment using the through-pores occurred more quickly than those through conventional pores. Although gas bubbles developed along the boundary between the CL and the PTL, they ended up migrating through pores toward the PTL surface [200]. Figure 15b depicts the movement of gas bubbles in the PTL and bubbles produced at the interface between the CL and the PTL.

When these bubbles merges with neighboring bubbles, its volume and surface energy are increased, and when it reaches the critical size, it breaks from the nucleation zone [141]. For an efficient gas bubble transfer, it is critical to minimize gas accumulation on the CL. This can be accomplished by employing a suitable PTL, which provides a conduit for gas bubbles to migrate away from the anode CL [204]. At a higher current supply, O_2_ gas bubbles generated in the PTL can block the flow of water toward the anode CL, resulting in water supply deficiency [25,205]. To effectively deliver water to the anode side, the PTL’s pores need to be larger, followed by a thicker catalyst coating [206]. The pore size and the thickness of the CL can significantly influence the PTL in a cell. As shown in Figure 15c, resistance increases with a decrease in the thickness of the CL because a thinner CL causes more restriction of electrons [201]. Additionally, Miličić et al. [202] reported that by increasing the liquid flow rate, the PTL liquid saturation is enhanced because of the efficient removal of gas bubbles from the PTL, which can reduce gas deposition in both the PTL and the anode CL, as presented in Figure 15d. The most common materials used for fabrication of the PTL on the anode side include Ti mesh, sintered powder, felt, multilayered, perforated plates, and others [127,144,207]. These materials have excellent chemical and mechanical stability [208]. Understanding the behavior of the bubble in the porous transport layer (PTL) becomes crucial to minimize voltage loss, increase efficiency, and improve the performance of the cell’s durability. Figure 15e depicts the growth and detachment of a single gas bubble in a channel at different times. A real-time study for gas bubbles showed that at time t = 0 ms, the bubble size diameter was 70 µm. By increasing the time t to 900 ms, the bubble size increased to 130 µm [203]. Understanding how gas bubbles travel in the PTL in real time can assist in determining factors that impact mass transport and optimize the PTL structure to improve system efficiency. Furthermore, analysis of how voltage fluctuates over time at a high current density has revealed that the commercial PTL experiences a quick increase in voltage due to inadequate removal of gas bubbles at reaction sites [144].

## 6. Measurement Techniques for Bubble Dynamics

There are various techniques available to measure the bubble size in a PEM water electrolysis system, including photography, neutron imaging, X-ray imaging, and acoustic emission. These methods are discussed as follows.

### 6.1. Photography Technique

**Figure 16:**
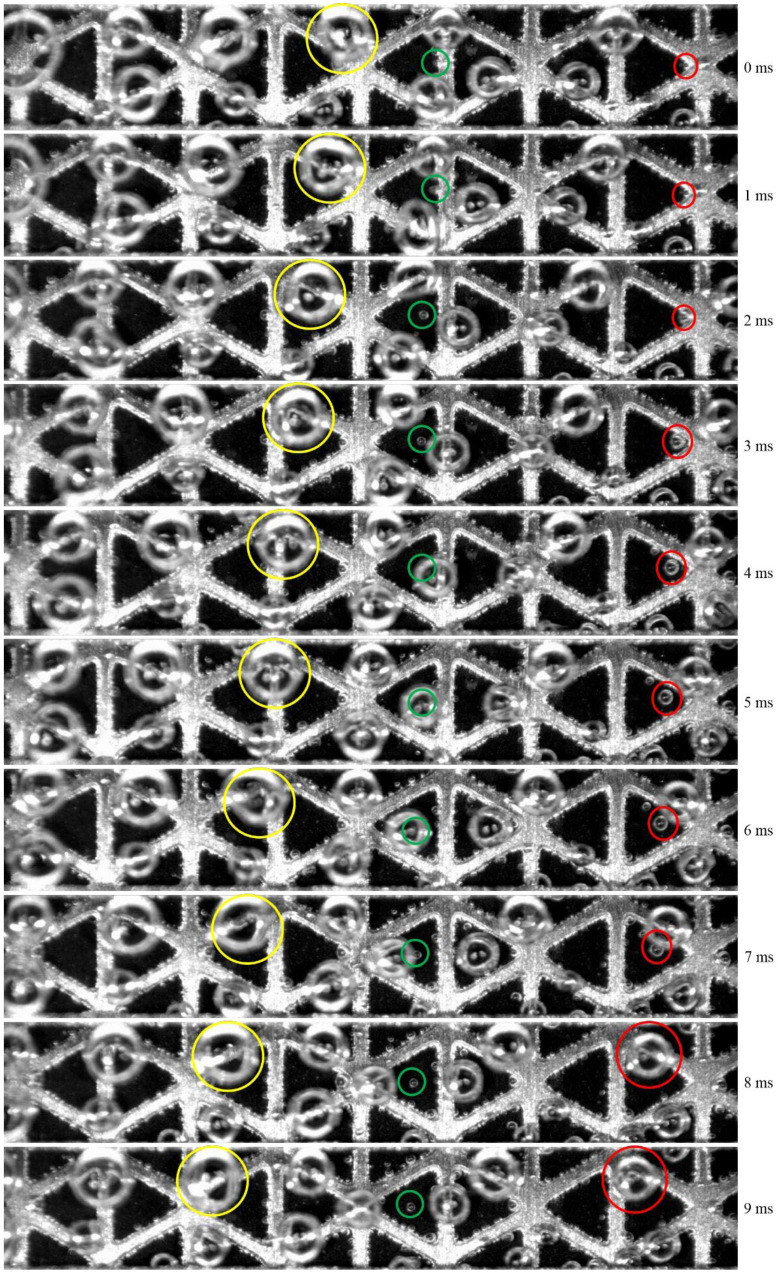
Captured bubble size image in PEM water electrolysis. Copyright Thesis 2016, with permission from Mo [35].

This technique is one of the simplest and most intuitive tools for measuring bubbles in a PEM water electrolysis system. Over the years, various studies have used both intrusive and nonintrusive methods to measure bubble size. In this methodology, the bubble sizes are measured using a photographic method followed by image analysis. Conventional microscopes or cameras cannot capture gas bubble dynamics at the microscale with ultra-high speeds. A high-speed camera is used to capture the different bubbles inside the PEM water electrolysis system since the bubbles generated in this type of instrument exhibit microbubble dynamics [111]. While using the high-speed camera, an LED light source is used for capturing the bubble images [38]. The light source is used to make moving bubbles more visible, preventing blurred motion and enabling accurate bubble behavior analysis. The image analysis process involves several processes, such as image segmentation, visibility adjustments, and contrast enhancement. Image analysis tools such as MATLAB, ImageJ, Adobe Reader, and Digimizer can be used to measure bubble size images. A captured bubble image from the PEM water electrolysis system using a high-speed camera is presented in Figure 16. The following expression can be used to measure the Sauter mean bubble diameter [209,210]:

(15) $$d_{32}=\frac{\sum_{i=1}^{n}n_{i}d_{d}^{3}}{\sum_{i=1}^{n}n_{i}d_{d}^{2}}$$

where *n_i_* denotes the number of bubbles with size diameter *d_d_*. For bubbles with an ellipsoidal shape, the bubble diameter can be measured using the following equation:

(16) $$d_{eq}=\sqrt[{3}]{l_{maj}^{2}l_{\min}}$$

where *l_maj_* and *l*_min_ are the major and minimum measured bubble lengths, respectively.

### 6.2. Neutron Imaging Technique

**Figure 17:**
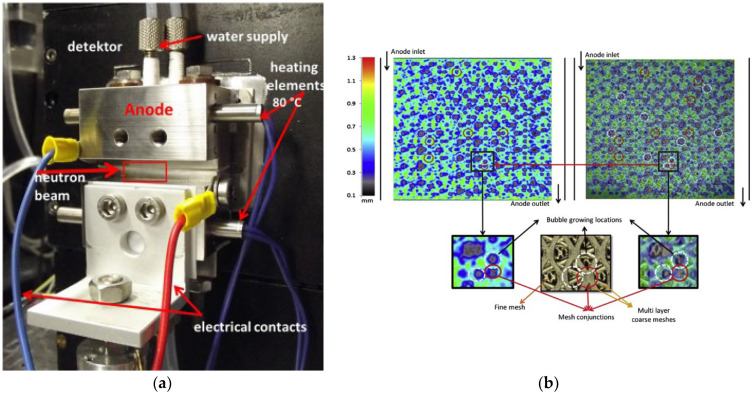
PEMWE neutron radiography. (**a**) Schematic image of neutron radiography. Copyright 2018, with permission from Elsevier [90]. (**b**) Integrated neutron-captured bubble image. The dotted circles represent stationary gas bubbles, whereas the solid circles depict increasing bubbles moving between holes. Copyright 2013, with permission from Elsevier [130].

In this method, images are recorded and the amount of water and gas in the porous layer is measured. Although the neutron imaging approach cannot provide clarity or detailed image quality like X-ray images, it has a significant advantage. Figure 17a shows neutron beam radiography images used for capturing bubble size while performing the experiment. It can penetrate through certain metals such as titanium, which is often used in electrolysis as a porous layer [211]. In neutron imaging, the PEM water electrolysis is initially placed at the neutron beam, and images are captured at a rate of 1 Hz. While experimenting, the neutron imaging failed to differentiate between gas bubbles and the titanium mesh used for the flow field. Therefore, combining both neutron and optical imaging techniques could help distinguish gas bubble evolution from the Ti mesh layers [130]. The intensity of the neutron is altered based on the presence of water inside the cell. Lee et al. [212] initially captured the reference image without applying a current under a fully saturated liquid in the anode when nitrogen gas is purged in the cathode side to remove the presence of water during the reference image capture. Finally, the image was captured by supplying the current in the electrolysis. An integrated bubble image is shown in Figure 17b. For image processing, three steps were used to measure the images [213]. While capturing images, random spots might be captured owing to changes in camera illumination. To eliminate these spots, the median value from multiple images is taken, yielding a sharper and cleaner image. The second step involves taking the median at every frame of three by three pixels to reduce electronic system noise [212]. Lastly, a procedure known as image restriction is adopted to avoid image alignment being disturbed during image capture due to changes in camera equipment alignment. These integrated approaches can improve our understanding of water behavior. They can be used to optimize PEM electrolysis performance. Another study used the same neutron imaging exposure duration of 5 s with two distinct types of images taken before the experiment. One is known as a dark field image (without using a neutron beam) and the other is described as a dry image (before circulating water inside the cell) [196]. Captured images were edited with ImageJ software and 30 images from each batch were merged using a process known as median averaging to further enhance the image quality. Real-time images from PEMWE were captured with a neutron beam and analyzed to determine water thickness using the Beer–Lambert law. The area known as land location was taken into consideration. The water within the land and in pore structures was then examined.

Using the Lambert–Beer Law, the thickness of water (*t*) in the region surrounding the bubble can be measured to comprehend and quantify its impact on the movement of water, which is expressed as [90,130]:

(17) $$t=\frac{-1}{n_{w}}\ln\left(\frac{I_{c}}{I_{d}}\right)$$

where *n_w_*, *I_c_*, and *I_d_* denote the number of neutrons absorbed or scattered by water during the process, the actual neutron image captured during the cell operation, and the neutron image recorded when the cell is completely dry.

### 6.3. X-ray Imaging Technique

**Figure 18:**
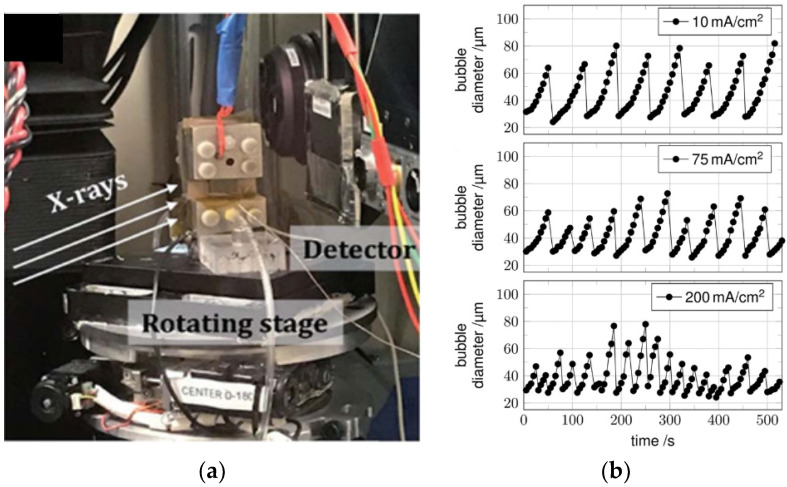
X-ray imaging setup. (**a**) Schematic diagram of the X-ray imaging in a PEM water electrolysis. Copyright 2020, with permission from the Royal Society of Chemistry [217]. (**b**) Formation of hydrogen bubble between PTL and cathode channel at different current densities. Copyright 2015, with permission from Elsevier [86].

This non-invasive method produces high-resolution 3D images. It has been used for measuring bubbles inside a PEM water electrolysis system. For studying the in-situ measurement of gas bubbles, different types of imaging such as optical, neutron, and X-ray imaging have been used [212,214]. This method is used as an ex-situ technique for assessing the physical structure and characteristics of materials such as the PTL in a water electrolysis system [215]. X-ray imaging and X-ray radiography have been employed to measure the presence of oxygen bubbles inside a PEM water electrolysis system, as shown in Figure 18a [203]. The setup was rotated 180°, and the tomography was allowed to scan from different angles by carefully selecting the beam energy for penetrating the system. Hoeh et al. [86] studied the evolution of gas in the PTL using the X-ray technique and analyzed a comprehensive review through plane and in-plane orientation. They used a concentrated beam of X-ray energy and allowed it to pass through the PEM water electrolysis system. Bubble images were captured using a CCD camera after every 5 s. They adopted a method called in-plane synchrotron radiography and determined gas bubbles between the PTL and flow channels. Figure 18b depicts how hydrogen bubbles change over time, indicating that higher current densities result in faster hydrogen bubble production and discharge, whereas lower current densities result in slower bubble growth. De Angelis et al. [216] have used the tomography method and studied how oxygen bubbles formed and interacted with or without the PTL structure. They observed bubble growth and detachment at the PTL, as well as the bubble rise velocity and change in behavior.

### 6.4. Acoustic Technique

**Figure 19:**
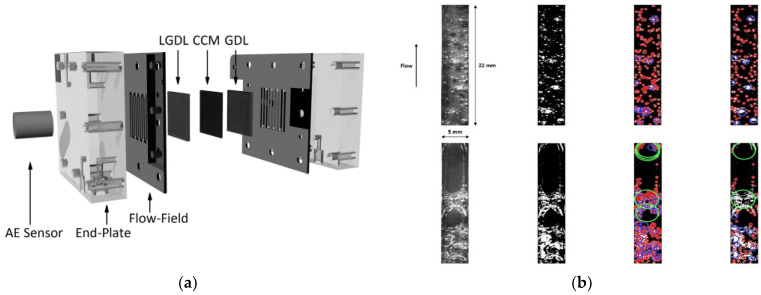
Schematic diagram of the PEMWE. (**a**) With an acoustic emission sensor. Copyright 2020, with permission from Frontiers [24]. (**b**) Captured bubble images using AE sensor. Copyright 2019, with permission from Elsevier [85].

Acoustic emission can be measured by using a cylindrical piezoelectric sensor at the anode side as shown in Figure 19a. In the HER process, a new design called surface-reflected bulk wave, which is a specific type of acoustic wave, was developed to prevent the accumulation of gas bubbles on the electrode, particularly at high current densities [218]. For operating the system, a constant current (galvanostatic) is applied, and the data collected every 1 min. Unlike other sensors, this acoustic emission (AE) can detect signals from all sides of the PEMWE cell. Further, collected data can be processed with AEwin software for enhancing the acoustic signal and for removing unwanted noise. During the operation, the system experiences mechanical perturbation due to the movement of bubbles, pressure fluctuation, and flow disturbance. These sensors can continuously generate voltage/time signals when perturbation waves are generated from the cell. With an increase in the physical vibration around the anode side, a sensor produces a high voltage in response. When the resulting voltage/time signal is higher than the desired noise threshold (set at 37 dB) for the purpose of detecting these AEs, it is referred to as an acoustic hit. While gathering data, each acoustic signal generates a distinctive sound. Four separate signals are generated, including frequency (hit rate), maximum sound wave volume (hit amplitude), time (hit duration), and energy (hit energy) produced with each impact. This can be used to understand whether the system has abnormal sound events or if there are any regions that need to be fixed while constructing the system. Thus, using this special sensor can monitor bubble behavior in the system. The impulse pressure generated by oscillating bubbles in the cell produces a certain sound frequency [219,220]. These oscillatory forces generated by vibration can trigger the shifting of the bubbles [221]. A captured bubble using the AE sensor is shown in Figure 19b. Larger bubbles have lower frequency oscillations, whereas smaller ones have higher frequencies. As a result, the velocity at which a bubble moves is influenced by its size. The relationship between bubble size and the frequency of the fluctuation has value for understanding bubble behavior in liquids. It potentially has several research applications [85]. For free oscillation of the bubble in the liquid, the relationship between the initial bubble size (*R*_0_) and the oscillating frequency (*f*) can be expressed as [222]:

(18) $$f=\frac{1}{2\pi }\sqrt{\frac{3kp_{\infty }}{\rho R_{0}^{2}}-\frac{2\sigma }{\rho R_{0}^{3}}}$$

where *p*_∞_ is the liquid pressure at a point distance from the oscillating bubble, *ρ* is the liquid density, *σ* is the liquid surface tension, and *k* denotes the polytropic coefficient. The relationship between the pressure (*P*) and the volume (*V*) during the growth or reduction of the gas bubbles is as follows [223]:

*PV^k^* = constant (19)

Similarly, the frequency oscillation of the bubble is expressed as [222,224]:

(20) $$f=\frac{1}{2\pi }\sqrt{\frac{3kp_{\infty }}{\rho R_{0}^{2}}}$$

This shows that the bubble frequency is inversely proportional to the bubble radius. Although bubbles in an electrolyzer flow channel may not be able to freely oscillate, bubble collisions may induce similar interactions between bubble size and frequency. Such relationships may provide knowledge regarding bubble dynamics in a flow channel, resulting in an impact on the operation of the whole system and bubble management.

## 7. Current Challenges and Outlook

PEM water electrolysis holds significant promise for producing the optimal green hydrogen. However, an in-depth understanding of the role of bubble dynamics in system performance is essential for solving the existing challenges before industrial-scale setups. In the literature, there are different studies carried out for the development of novel on the development of novel catalysts, bipolar plates, flow channels, and PTL materials. All these investigations have significantly enhanced the system performance, but challenges remain, particularly the controlling of bubbles across the electrode surface and from the flow channel. Different studies have been carried out in the context of catalyst coatings for the enhancement of the OER and HER through nanostructures, which have improved the bubble evolution [190,225]. However, it is still not identified how these nanostructures impact the bubble evolution. One such persistent problem is the effective removal of bubbles from electrode surfaces, which necessitates higher current densities, lowering system efficiency significantly. When these bubbles build up on the electrode surface, they can obstruct the pores, which in turn causes activation losses and mass transport losses [23,226,227]. Another key step is to identify hotspots for bubble generation on electrode surfaces. Bubble evolution may vary for electrode surfaces with different pore diameters. Thus, identifying these hotspots is critical for the timely removal of oxygen gas bubbles. Therefore, future research should emphasize the detailed investigation of how these bubbles cover the electrode surfaces to improve gas bubble removal and minimize system damage. Flow channel rib pressure may destabilize the PTL layer, preventing proper gas flow in the region. It is still unclear how the flow channel’s rib affects the PTL layer. Future studies ought to focus on the complex relation between flow channels, ribs, and PTL layers to optimize gas distribution and enable effective electrolysis. More experimental and modeling research is needed to comprehend the role of bubble evolution in overcoming present challenges in industrial-scale setups. This will bridge the gap between laboratory findings and real-world applications. The present review paper lays the foundation for future research initiatives aimed at overcoming current barriers and advancing PEM water electrolysis.

## 8. Conclusions

Hydrogen gas is regarded as one of the greenest and most sustainable energy sources. It has the potential to replace traditional fossil fuels in various applications such as automobile fuel, energy generation, heating, industrial operations, and so on. Using electrical energy, PEM water electrolysis can produce green hydrogen gas by simply splitting water molecules into oxygen and hydrogen. Despite significant advances in hydrogen gas production via water electrolysis, there are still numerous obstacles in predicting and managing bubbles inside a cell. Although innovative electrode design can provide efficient bubble control in the cell, there is a main issue associated with the cost of various components. This article presented a comprehensive review based on how the bubbles stick on the electrode surface and its substantial impact on cell performance, resulting in various losses in the system. It also addressed the effectiveness of PEM water electrolysis which can be greatly impacted by interactions between activation overpotential, ohmic overpotential, and diffusion overpotential due to mass transport losses. Overpotential losses can be minimized by lowering bubble buildup on the electrode surface, which in turn can improve ion flow and mass transfer efficiency. Both bubble evolution and transport must be enhanced to control bubbles by improving the porous transport layer and catalyst coatings. Efficient bubble dynamics and the different stages of a bubble from nucleation to detachment were discussed. Increased bubble nucleation can generate a swarm of small bubbles on the CL, which can enhance mass transport, promote gas evolution, and decrease the formation of large bubbles. This paper also focused on the complex bubble dynamics inside a flow channel, a catalyst layer, and a porous transport layer. It highlighted the crucial function of low pressure drop, restricted bubble accumulation, and precise ionomer control in the CL, underlining their ability to prevent bubble buildup. Furthermore, it demonstrated that bubbles can be swiftly removed when PTL structures are designed with appropriate pore size and thickness. In addition, measurement techniques for various methods of measuring bubble size in a PEMWE cell were explained. The incorporation of accurate measuring techniques enhances a thorough understanding of bubble behavior, enabling the development of more efficient and optimal PEMWE systems.

## Figures and Tables

**Table 1:** Advantages and disadvantages of PEM water electrolysis and PEM fuel cells [47,48,49].

Characteristic	PEM Fuel Cell	PEM Water Electrolysis
Advantage		
Energy efficiency	Production of power from hydrogen and oxygen supply.	Utilized renewable energy to produce hydrogen gas.
Low environment impact	Only produces water as a byproduct.	Oxygen as a byproduct.
Usage	Used in various applications like transportation and power sources.	It can be used for large-scale hydrogen production.
Start-up process	Ability to start and stop quickly, making it viable for different power demands.	Fast response and simple control.
Disadvantage		
Higher cost	Catalysts like Pt are expensive, which increases production costs.	PEM membrane manufacturing requires expensive components and infrastructure.
Catalyst shortage	Catalysts require metals like Pt, resulting in supply concerns.	Catalyst materials are constrained, which affects both cost and efficiency.
Longevity and stability	Catalyst’s lifespan decreases over time.	Gradual degradation of the catalyst’s membrane impacts its durability.

**Table 2:** Different key components used in PEM water electrolysis.

Anode- and Cathode-Side Current Distributor	Anode-Side Catalyst	Cathode-Side Catalyst	Catalyst-Coated Membrane	Cathode-Side Diffusion Layer	Cell Area	Bipolar Plate	Flow Pattern	Authors
Ti	2.3 mg/cm^2^ IrO_2_	1.0 mg/cm^2^ carbon-supported Pt	Nafion-115 and Nafion-117	Sigracet^®^ 28BCE carbon and Ti	25 cm^2^	Ti	Parallel flow	[82]
Ti	3.0 mg/cm^2^ Ir/RuO_x_	0.6 mg/cm^2^ Pt	Nafion-115	Toray H-060 carbon paper	9 cm^2^	-	Parallel flow	[24]
Ti mesh and carbon paper (type 34BA)	1.5 mg/cm^2^ IrO_2_	0.5 mg/cm^2^ Pt (46 wt% Pt/C)	Nafion^®^ 117	-	64 cm^2^	-	Parallel flow, serpentine flow, cascade flow	[83]
Ti	1.0 mg/cm^2^ Pt	1.0 mg/cm^2^ Pt	Nafion	-	25 cm^2^	Standard graphite	Parallel	[80]
Pt-plated Ti (anode) and carbon (cathode)	IrO_x_	Pt/C	Nafion-115	Toray H-060 carbon paper	-	-	-	[84]
Gold-coated Ti (anode) and Cu (cathode)	2.0 mg/cm^2^ Ir/IrO_x_	1.0 mg/cm^2^ Pt black	Nafion-117	Toray 090 carbon paper	5 cm^2^	Graphite	Parallel	[30]
-	3.0 mg/cm^2^ Ir/RuO_2_	0.6 mg/cm^2^ Pt	Nafion-115	Sintered Ti powder	13.5 cm^2^	-	Single flow	[85]
Ti (anode) and carbon (cathode)	IrO_2_	Pt/C	Nafion-117	Toray H 120 carbon paper	11.9 and 17.6 cm^2^	Graphite	Parallel and meander shaped single channel	[86]
Ti (cathode)	3.0 mg/cm^2^ IrRuO_x_	3.0 mg/cm^2^ Pt black	Nafion-115	Toray 090 carbon paper (anode side)	5 cm^2^	-	Parallel flow	[5]
Carbon paper, fine Ti meshes and sintered porous Ti	2.0 mg/cm^2^ Pt and 2.0 mg/cm^2^ Ir	4.0 mg/cm^2^ Pt	Nafion-110	-	50 cm^2^	Ti	Mesh	[87]
-	2.0, 0.3, 3.0, 1.0 mg/cm^2^IrO_2_/Ir/Ir black	0.8, 0.5, 3.0, 0.9 mg/cm^2^ Pt and Pt/C	Nafion-115	-	120 cm^2^	-	-	[21]
-	3.0 mg/cm^2^ IrO_2_	1.0 mg/cm^2^ Pt	Nafion-117	Sintered porous Ti plate	25 cm^2^	Ti	Parallel flow	[88]
Ti and gold-plated	1.0 mg/cm^2^ Ir	0.3 mg/cm^2^ Pt	Nafion-HP	Sintered Ti powder	0.8 cm^2^	Ti	Parallel flow	[89]
-	2.2 mg/cm^2^ Ir	0.8 mg/cm^2^ Pt	Nafion-117	Toray H 120 carbon paper	1.5 cm^2^	-	Parallel and interdigitated flow	[90]
Ti and gold-plated	1.0 mg/cm^2^ Ir	0.3 mg/cm^2^ Pt	Nafion-1110	Toray H 060 carbon paper	0.8 cm^2^	-	Parallel flow	[91]

## Data Availability

Not applicable.
